# A simple approach to spectrally resolved fluorescence and bright field microscopy over select regions of interest

**DOI:** 10.1063/1.4967274

**Published:** 2016-11-29

**Authors:** Peter D. Dahlberg, Christopher T. Boughter, Nabil F. Faruk, Lu Hong, Young Hoon Koh, Matthew A. Reyer, Alon Shaiber, Aiman Sherani, Jiacheng Zhang, Justin E. Jureller, Adam T. Hammond

**Affiliations:** Graduate Program in the Biophysical Sciences, Institute for Biophysical Dynamics, and The James Franck Institute, The University of Chicago, Chicago, Illinois 60637, USA

## Abstract

A standard wide field inverted microscope was converted to a spatially selective spectrally resolved microscope through the addition of a polarizing beam splitter, a pair of polarizers, an amplitude-mode liquid crystal-spatial light modulator, and a USB spectrometer. The instrument is capable of simultaneously imaging and acquiring spectra over user defined regions of interest. The microscope can also be operated in a bright-field mode to acquire absorption spectra of micron scale objects. The utility of the instrument is demonstrated on three different samples. First, the instrument is used to resolve three differently labeled fluorescent beads *in vitro*. Second, the instrument is used to recover time dependent bleaching dynamics that have distinct spectral changes in the cyanobacteria, *Synechococcus leopoliensis* UTEX 625. Lastly, the technique is used to acquire the absorption spectra of CH_3_NH_3_PbBr_3_ perovskites and measure differences between nanocrystal films and micron scale crystals.

## I. INTRODUCTION

Optical microscopy is a powerful tool across all scientific disciplines. Advances in instrumentation are occurring rapidly, with much of the current efforts concentrating on increasing spatial resolution beyond the diffraction limit in three dimensions.^1–7^ However, microscopy instrumentation also benefits from the addition of other types of information, including temporal and spectral information. Spectrally resolved fluorescence microscopes, sometimes called hyperspectral fluorescence microscopes, are capable of acquiring an accurate spectrum of the emitted light and are generally found in high-end laser scanning confocal microscopes or systems with specialized cameras.^8–11^

Access to spectral information offers additional experimental capabilities for a standard fluorescence microscopy. A common application of fluorescence microscopy is imaging fluorescently labeled proteins. Introducing spectral information allows for discrimination between numerous fluorescent labels. Traditionally, the fluorescent signal from any specific fluorophore is separated from the excitation spectrum using optimized filter sets.^12^ Biological systems, however, often involve numerous proteins working in tandem, necessitating the simultaneous imaging of multiple proteins with different fluorescent labels.^13^ Significant work has been done to design fluorophores that have distinct emission spectra^14^ and instruments have been constructed that use multiple bandpass filters and cameras for simultaneous imaging of multiple fluorophores.^15^ Even with an optimized choice of fluorophores and filters, cross talk remains a concern.^12^ While spectral sampling methods such as linear unmixing are powerful, the acquisition of complete fluorescence spectra allows for an unambiguous separation of spectral features and, if desired, an accurate quantification of spectral overlaps.

Beyond separating different spectral components, spectrally resolved fluorescence signals can be used as a reporter of the local environment surrounding fluorescent labels. Fluorescence spectra and lifetimes are influenced by pH, ionic concentration, membrane potential, solvation, and even the presence of a magnetic field.^16–18^ The techniques for measuring these phenomena in bulk samples are well established, and become accessible for microscopy when similar spectral resolution can be obtained.

Equally important to spectral resolution in light microscopy is the ability to spatially limit the spectral analysis to a region of interest (ROI) within the field of view. In this paper, we use a Spatial Light Modulator (SLM) to select these regions. SLMs are optical components that can manipulate the phase and/or amplitude of incident light.^19^ There are numerous technologies that are classified as SLMs. The most common forms of SLMs are deformable mirrors, digital micro-mirror devices, and liquid crystal SLMs (LC-SLMs). Initially, SLMs were developed for video projection but they have found widespread use in numerous fields including optical microscopy. SLMs are used in both the illumination and imaging paths of microscopes.^12,19^ When placed in the illumination path, SLMs are commonly used to produce spatially structured illumination at the sample position and to generate structured wavefronts. When placed in the imaging path, SLMs are often added to the Fourier plane. This plane represents the spatial Fourier transform of the sample imaging plane.^12,20,21^ When placed here they can be used to manipulate the phase or amplitude in Fourier space. This has been used to achieve nanometer scale precision in *z* by changing the point-spread function, recover molecular orientation, and enhance phase contrast microscopy.^1–3^

In this manuscript we outline the addition of an amplitude-mode LC-SLM and a spectrometer to a secondary image plane (rather than a Fourier plane) that allows for a standard inverted microscope to simultaneously acquire diffraction limited fluorescence images and fluorescence spectra of user-defined arbitrary ROIs. The same instrument, when operated in bright-field transmission mode, can be used to simultaneously acquire bright-field images and absorption spectra of micron scale objects or ROIs. The ability to acquire absorption spectra of microscopic objects is a significant advantage over existing hyperspectral microscopes, which typically use laser scanning monochromatic excitation sources that are impractical for spectral absorption measurements. Measuring the absorption spectra of micron scale objects can be difficult in practice and is usually achieved using a small pinhole in standard UV-VIS spectrometers or using specialized custom instrumentation.^22^ This requires the precise placement of the object in relation to the pinhole making the creation of a spatial map of a sample difficult and tedious.

To demonstrate the utility of this design, we apply this microscope to three different samples. First, we use the microscope to resolve three differently labeled fluorescent beads mixed on a standard microscope slide. Second, we show time dependent bleaching dynamics that have distinct spectral changes in the cyanobacteria, *Synechococcus leopoliensis* UTEX 625. Lastly, we use the microscope to acquire absorption spectra of CH_3_NH_3_PbBr_3_ perovskites and show differences between nanocrystal films and micron scale crystals.

## II. MATERIALS AND METHODS

### A. Spatially selective spectrally resolved microscope

**FIG. 1.**
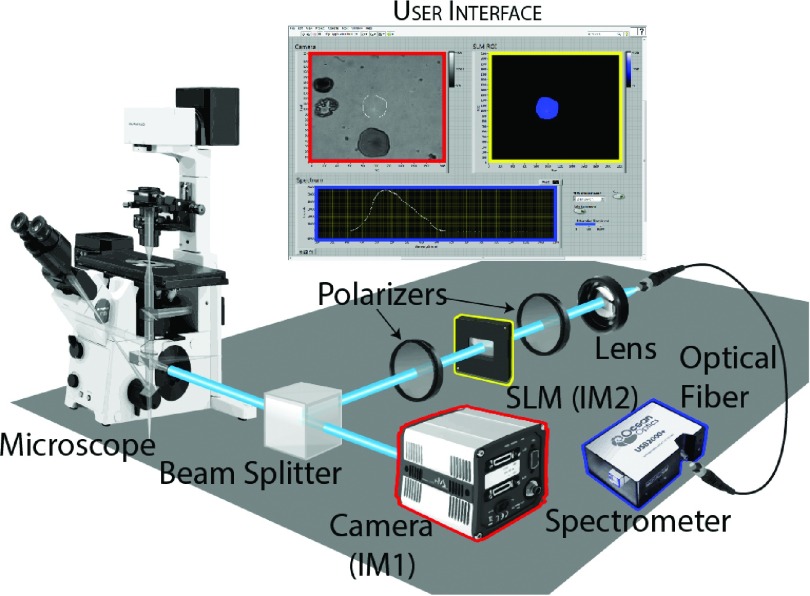
Diagram of the spatially selective spectrally resolved microscope. The polarizing beamsplitter at the side port of the microscope splits the normal image plane formed by the tube lens into two separate image planes. At one plane a monochrome camera is placed to collect standard microscope images. At the other plane a LC-SLM is placed where an ROI can be selected in reference to the camera image. Light in this ROI is then spectrally resolved by the fiberoptic spectrometer. The microscope is controlled via a user interface programmed in LabVIEW that coordinates the camera, spectrometer, and SLM.

**FIG. 2.**
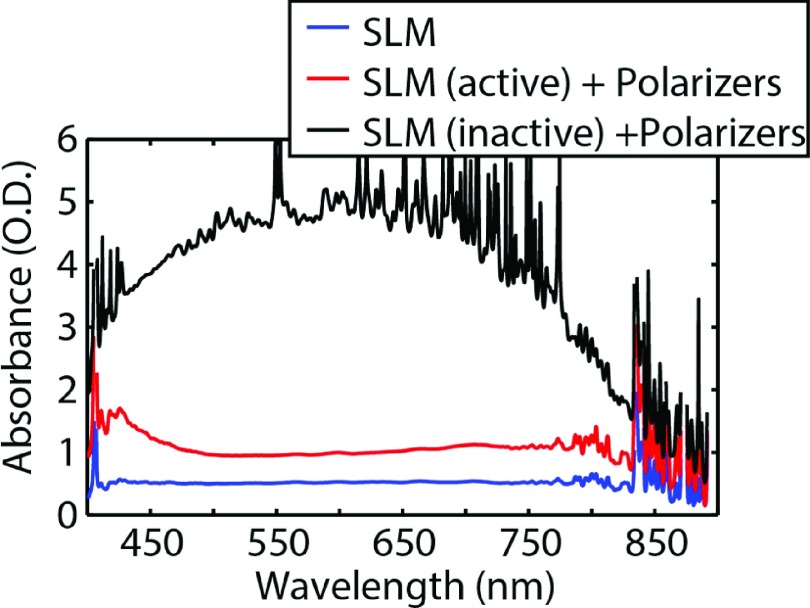
Controlled suppression of light by the SLM/polarizer optical system. The blue trace shows the absorption that occurs just with the SLM. The red trace shows the absorption through the optical path of S-polarizer → LC-SLM → P-Polarizer when the LC-SLM is actively rotating the polarization of all pixels by 90°. The black trace is the same as the red except the LC-SLM is inactive, thus minimizing the transmitted light.

Figure 1 shows a diagram of the microscope. The microscope is a standard inverted microscope (Olympus IX71) and none of the internal components have been altered. A broadband polarizing beam splitting cube (Thorlabs PBS253) is placed directly (∼2 cm) after the side port of the microscope and produces two image planes from the tube lens in the microscope, IM1 and IM2, at a distance of ∼15 cm from the side port. At IM1 a CMOS camera (Andor Zyla 4.2) acquires standard fluorescence images, while an amplitude-mode transmissive LC-SLM (Holoeye LC 2002) is placed at IM2 between two crossed polarizers. The LC-SLM is used in amplitude mode to select ROIs from the fluorescence image. The polarizer closest to the beam splitter is aligned to transmit the maximum amount of light, i.e., aligned with the optical axis of the polarizing beam splitting cube. When an LC-SLM pixel is inactive the second polarizer blocks light from that pixel, and when an LC-SLM pixel is active the polarization is rotated 90° allowing the light from that pixel to be transmitted through the second polarizer, see Figure 2. Light that passes through the second polarizer is collected by an aspheric lens with a focal length of 16 mm (Thorlabs ACL25416U) and focused onto an optical fiber (600 μm diameter multimode fiber Thorlabs M34L01) coupled to a USB spectrometer (Ocean Optics USB 2000). A spatial mapping of pixels between the LC-SLM at IM2 and the camera at IM1 was achieved by using an additional camera and lens system to image the LC-SLM at IM2 while scanning the edge of a razor blade across the field of view along the horizontal and vertical axis. Comparing the razor’s position at several points on the LC-SLM at IM2 and on the camera at IM1 yielded a linear relationship that mapped the pixels on the LC-SLM to the pixels on the camera at IM1. This mapping allows for the drawing of an arbitrary ROI based off of the fluorescence image to be mapped onto the LC-SLM. The pixels in the ROI on the LC-SLM rotate the polarization of transmitted light by 90°, allowing only light that passed through the ROI to be transmitted through the second polarizer and into the spectrometer. Software with a graphical user interface made in LabVIEW acquires the images, allows the user to select pixels that define arbitrary ROIs, and collects the spectra. Sample illumination is either performed using a tungsten halogen lamp (Olympus U-2H100L-3) in bright-field trans illumination or using a white light LED (Thorlabs MCWHL2-C1) filtered with a narrow band pass excitation filter centered at 450 nm during epi-fluorescence imaging. A long pass emission filter and dichroic with a cutoff at 530 nm was used to exclude the excitation wavelengths. All acquired spectra were convoluted with a Gaussian to minimize electrical noise and reflect the optical resolution of the spectrometer, which was calculated to be ∼3.5 nm based on the entrance slit (100 μm) and dispersion of the 600 line/mm grating. The spectrometer was calibrated using a neon lamp (Pen-Ray, Inc.), see Figure S1 of the supplementary material. The lateral resolution of the instrument differs only slightly from that of a standard wide field inverted microscope. The point-spread function for the fluorescence images is slightly astigmatic with a FWHM lateral resolution of 246 nm in the vertical and 349 nm in the horizontal measured with a 100 × 1.3 NA plan-fluor oil immersion objective (Olympus UPlanFLN) and a 1.3 × magnification changer, see Figure S2 of the supplementary material. The astigmatism is possibly due to focusing through the polarizing beam splitting cube or the polarization selectivity biasing the camera image to fluorophores in certain orientations.^23^ The resolution of the SLM differs from the fluorescence image only in that the SLM pixels are ∼5 times larger in each dimension, see Figure S3 of the supplementary material.

### B. Sample preparation

#### 1. Fluorescently labeled beads

Three varieties of fluorescently labeled polymer microspheres were purchased from Cospheric LLC (FMY-1.3, FMOY-1.3, and FMO-1.3). The polymer spheres were a mixture of sizes from 1.5 to 5 μm in diameter. Each label was excitable at 450 nm but yielded distinct emission spectra in the range of 500-650 nm. For analysis, 50 *μ*g of each type of dry microspheres were diluted in 10 ml of water. Then, 20 *μ*l of this solution was placed on a standard slide and coverslip.

#### 2. Synechococcus leopoliensis UTEX 625

*S. leopoliensis*, a species of cyanobacteria, was grown according to standard protocols.^24^ Briefly, the cultures were grown autotrophically on rotary shakers under constant illumination of ∼110 *μ*E m^−2^ s^−1^ in 5.0% CO_2_.^31^ The cultures were grown in 250 ml culture flasks and the temperature was held between 25 and 28 °C. One day prior to the analysis 50 ml of the culture was removed and placed in the dark at room temperature. For analysis, a sample of the culture was placed between a standard slide and coverslip. All measurements were performed within 30 min of slide preparation.

#### 3. *CH_3_NH_3_PbBr_3_* perovskite microcrystals

The CH_3_NH_3_PbBr_3_ perovskite thin film preparation is adapted from the protocol in the work of Grancini *et al.*^25^ 40 wt. % of CH_3_NH_3_Br and 40 wt. % PbBr_2_ were dissolved in 2 ml of DMF. The solution was then filtered in a 0.2 μm syringe filter unit followed by spin-coating (2000 rpm for 60 s) on a 1 in. by 1 in. O_2_-plasma pretreated glass slide. The resulting film was then annealed at 150 °C for 1 h and stored under vacuum before use. This preparation resulted in a sparse heterogeneous sample that was a mixture of small micron scale crystals and “amorphous” films, which were likely composed of nanometer scale crystalline domains.

## III. RESULTS AND DISCUSSION

### A. Fluorescently labeled beads

**FIG. 3.**
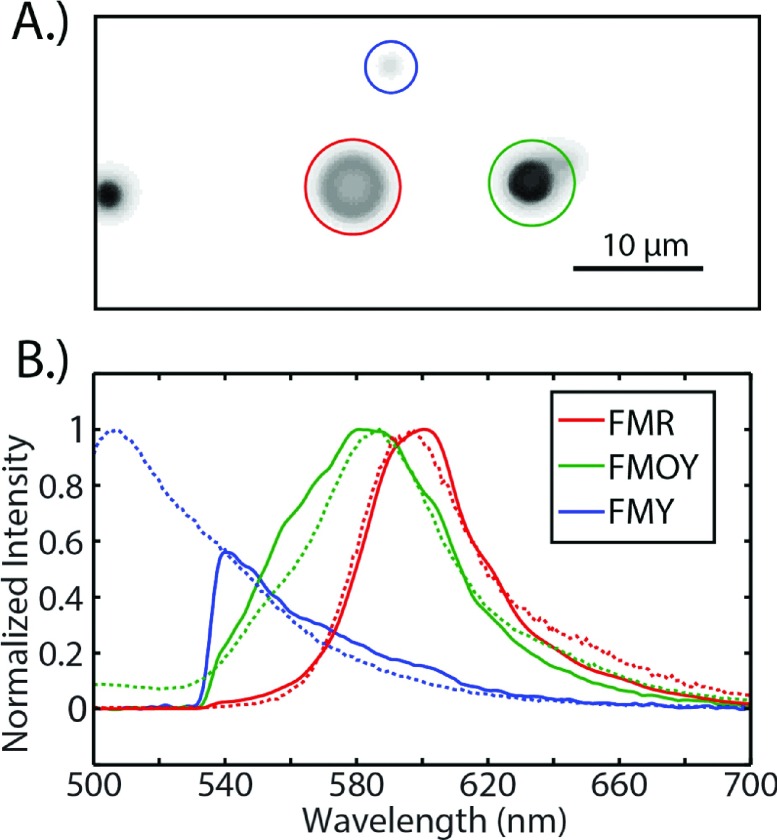
(a) Microscope image of the fluorescently labeled microspheres shown in inverted intensity. The three colored circles indicate the ROIs mapped to the LC-SLM. (b) The fluorescence spectra corresponding to the ROIs are shown in the solid lines. The dashed lines are fluorescent spectra of the bulk microspheres taken in a standard fluorometer under 450 nm excitation. The cutoff at 530 nm is caused by the dichroic in the filter cube.

The solution of fluorescently labeled microspheres, as prepared above, was imaged at 1 Hz and 130 × magnification with a 100 × plan-fluor oil immersion objective and 1.3 × magnification changer, see Figure 3. Fluorescence spectra of ROIs were acquired at 1 Hz as the ROI was changed to select different microspheres in the field of view, see Movie S4 of the supplementary material. Fluorescence images were acquired simultaneously with the individual spectra. The recorded fluorescence spectra of the three microspheres are shown in Figure 3 (solid lines). The frame rate of 1 Hz is much slower than the absolute temporal resolution achievable by the instrument. The ability to define distinct ROIs is limited by the 60 Hz frame rate of the SLM and the spectrometer can be run at 1 kHz if the source is bright enough to achieve a reasonable signal to noise ratio, see Figure S5 of the supplementary material. The fluorescence spectra taken with the microscope are compared to the fluorescence spectra acquired from solutions of the bulk microspheres taken in a fluorometer (Horiba Fluorolog-3) under 450 nm excitation (dashed lines). Despite the strong spectral overlap of the three different labels, the spectra are easily distinguishable and are in agreement with the fluorometer-obtained spectra from bulk. The differences between the spectra obtained in the microscope and those obtained using the fluorometer are likely due to differences in the instrument responsivity (e.g., grating reflectivity, detector response, objective transmission, and filter transmission) as well as photobleaching and photoconversion of the different dyes. The latter effect was most pronounced in FMOY. Over the course of several minutes, the fluorescence maximum of FMOY shifted by more than 50 nm, see Figure S6 of the supplementary material.

### B. Observation of photoprotective mechanisms in single cells

All photosynthetic organisms have a set of pigment-protein complexes that are responsible for the absorption of solar radiation, which are known as the photosynthetic antenna.^26^ These complexes transfer energy to a pigment-protein complex known as the reaction center, which is responsible for charge separation. Under high-light conditions, charge separation and subsequent electron transfer processes are unable to keep up with incoming excitations and if unquenched, these excitations can generate hazardous reactive oxygen species. Photosynthetic organisms employ numerous photoprotective mechanisms that aim to quench excitations under high-light conditions. These mechanisms are broadly referred to as non-photochemical quenching (NPQ) and are characterized in part by a decrease in the fluorescence intensity of the reaction centers.^27^ Here we use a 450 nm excitation to induce NPQ in *S. leopoliensis* and follow the process in time through changes in the fluorescent spectrum.

**FIG. 4.**
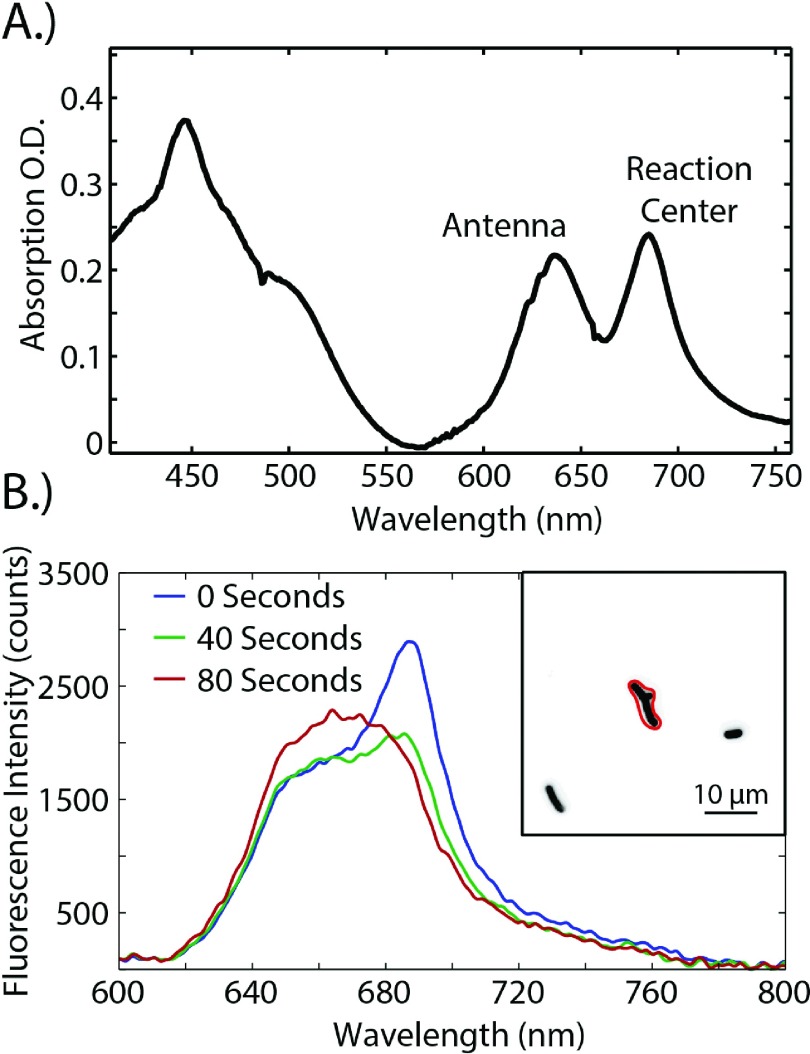
(a) Absorption spectrum of *S. leopoliensis* cell cultures after correcting for scatter contributions by the subtraction of a polynomial fit to long wavelengths taken in a standard UV-VIS spectrometer. The two peaks on the red side of the spectrum correspond to the photosynthetic antenna and the reaction center. (b) The inset shows a group of cells analyzed with the ROI shown by the red outline. The graph shows the corresponding fluorescence spectra for these cells as a function of time where 0 s corresponds to the first frame after turning on a 450 nm excitation.

The absorption spectrum of *S. leopoliensis*, taken with a standard UV/VIS spectrometer, can be seen in Figure 4(a). The two features on the red side of the spectrum, at 640 and 680 nm, correspond to absorption by the photosynthetic antenna and the photosynthetic reaction center, respectively.^28^ The fluorescence of the antenna is peaked around 660 nm and the fluorescence from the reaction center is peaked around 685 nm. Figure 4(b) shows the fluorescence spectra from a small group of cells as a function of time. Immediately upon the intense excitation the *S. leopoliensis* begins undergoing NPQ, see Movie S7 of the supplementary material. The series of fluorescence spectra show the rapid bleaching of the fluorescent feature at 685 (corresponding to the reaction centers) and a slight growth of the 660 nm feature (corresponding to the antenna). These dynamics are indicative of NPQ.^29^

### C. Absorption spectra of perovskite microcrystals

In the recent years, hybrid perovskites, of the form CH_3_NH_3_PbX_3_ [X = Cl, Br, or I], have gained widespread attention as light absorbers in the solar harvesting community. This attention is spurred by a remarkable device performance of perovskite solar cells with certified power conversion efficiencies as high as 20.1%.^30^ These materials are advantageous in part because of their solution phase processability. Solutions of hybrid perovskites are usually deposited on a conducting layer such as TiO_2_, forming a polycrystalline thin film. It has been shown that hybrid perovskites with small grain size (tens of nanometers) do not support excitonic states and only produce free carriers. However, free carriers in large crystals (hundreds of nanometers) thermalize and form excitonic states. This results in a red shifted absorption spectrum for large crystals relative to small crystals. This shift is arduous to measure using traditional spectrometers because the populations of crystals have to be purified separately.^25^

**FIG. 5.**
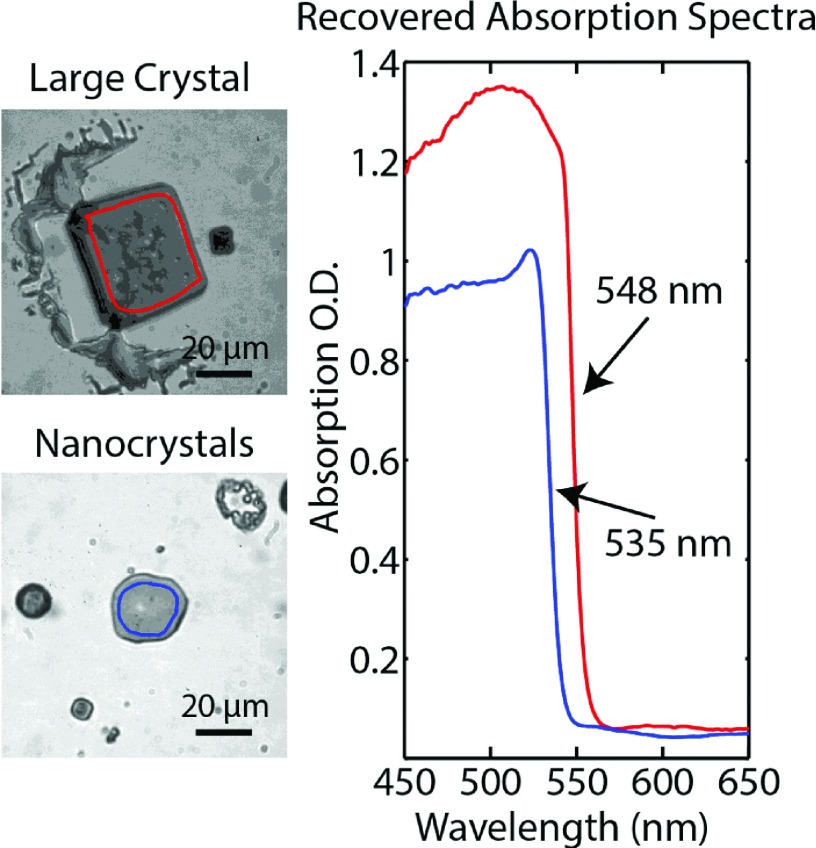
Microscope images of a large perovskite crystal and a film of perovskite nanocrystals. The absorption spectrum of the region corresponding to the red and blue outlines in the microscope images shows the expected redshift of the large crystal relative to the nanocrystal film.

Using bright-field illumination we can recover this shift in the absorption spectrum from a single sample containing both micron scale crystals and nanocrystal films. Absorbance is defined as

$$A\left(\lambda \right)=-\log_{10}\left(\frac{I_{i}\left(\lambda \right)}{I_{t}\left(\lambda \right)}\right),$$

where *I_i_*(*λ*) is the intensity of the incident light at a given wavelength, *λ*, and *I_t_*(*λ*) is the intensity of transmitted light.^26^ In order to obtain absorption spectra, $I_{t}\left(\lambda \right)$ is first measured by selecting an ROI centered on the sample of interest, then the sample is moved to the side of the ROI to measure *I_i_*(*λ*), see Movies S8 and S9 of the supplementary material. The log ratio of these two spectra gives the absorption spectrum. Figure 5 shows microscope images and absorption spectra taken with a 20× objective of a film of CH_3_NH_3_PbBr_3_ perovskites prepared in the manner described above. The shift between the two structures is ∼13 nm and is similar to the published shifts observed in CH_3_NH_3_PbI_3_ perovskites.^25^

## IV. CONCLUSION

Through the addition of a polarizing beam splitter, two polarizers, an amplitude-mode LC-SLM, and a USB spectrometer, we have converted a standard inverted microscope into a spatially selective spectrally resolved microscope. The microscope can simultaneously image and acquire spectra from an arbitrary, user-defined ROI. We have applied this technique to three different samples. The first was a mixture of fluorescent microspheres, which demonstrated the ability to separate congested fluorescent spectra and identify fluorophores with overlapping emission spectra. Second, we used the microscope to observe time-resolved photoprotective mechanisms in cells of cyanobacteria, demonstrating the ability to follow shifts in the fluorescence spectra with time. Lastly, we used the microscope to measure the redshift in absorption between large and small perovskite crystals, demonstrating the ability to rapidly measure absorption spectra of micron scale structures.

Future improvements and additions to the microscope could expand its capabilities. Simple changes such as a more broadband bright-field illumination source would allow for absorption spectra across a larger bandwidth. Similarly, a long pass emission filter more closely matched to the excitation spectrum would allow broader fluorescence spectra to be acquired. Also, spectral acquisition could be much faster with a more sensitive spectrophotometer. More complex additions could also be made. For example, instead of spectrally resolving the light transmitted through the LC-SLM one could imagine using a pulsed excitation source and replacing the spectrometer with time-correlated single photon counting hardware for lifetime measurements of the fluorescence signal or even performing ultrafast transient absorption measurements in a microscope on only a select ROI. The ability to spatially filter light from a secondary image plane expands the potential of a standard inverted microscope and provides a simple way to gain new insight into the microscopic world.

## SUPPLEMENTARY MATERIAL

See supplementary material for movies displaying data acquisition, and figures displaying the spectrometer calibration procedure, spatial and temporal resolution of the instrument, and the photoconversion of FMOY labeled microspheres.
